# Time optimization of ^90^Sr determinations: sequential measurement of multiple samples during decay of ^90^Y

**DOI:** 10.1007/s10967-016-5062-4

**Published:** 2016-10-07

**Authors:** Stina Holmgren Rondahl, Annika Tovedal, Oscar Björnham, Henrik Ramebäck

**Affiliations:** 1 CBRN Defence and Security, Swedish Defence Research Agency (FOI), Cementvägen 20, 901 82 Umeå, Sweden; 2 Department of Chemistry and Chemical Engineering, Nuclear Chemistry, Chalmers University of Technology, Kemivägen 4, 412 58 Göteborg, Sweden

**Keywords:** ^89^Sr, ^90^Sr, ^90^Y, MDA, Detection limit, Interferences, Optimization

## Abstract

This work presents an optimized method for the determination of multiple samples containing ^90^Sr when its daughter ^90^Y is measured after chemical separation and in sequence, *i.e.* during its decay. Consequently the measurement times will increase for each subsequent sample, since there has been a longer time for decay before measurement. Compared to a previously published approach, when ^90^Y is measured during its ingrowth, the gain in total analysis time (time for ingrowth+ summation of measurement times) is not that large, particularly not for low background instruments. However, results for a large part of the samples can be delivered earlier.

## Introduction

Rapid measurement of one of the most hazardous fission products, radioactive strontium, within a couple of months of *e.g.* a reactor accident will need a thorough consideration of potential interferences. This is especially important as one relatively long lived radioisotope, ^89^Sr (*t*_½_ = 54 days), will interfere when determining the other strontium isotope of interest ^90^Sr (*t*_½_ = 28.8 years), via measurement of the daughter nuclide ^90^Y (*t*_½_ = 64 hours).

When determining ^90^Sr there are a multitude of different methods presented in the literature [1–11]. A large part of the methods described in the literature consist of isolating strontium by chemical separation, in order to get samples free from interfering radionuclides, followed by spectrometric measurement of the emitted beta radiation [12–16]. Another approach is to make use of the fact that the daughter nuclide of ^90^Sr, ^90^Y, is a high energy beta emitter. It is therefore possible to measure ^90^Y by Cherenkov counting, a measurement approach discriminating towards low energy beta radiation (energy threshold in water is 0.263 MeV). A third approach is to measure ^89^Sr by Cherenkov counting and ^89^Sr–^90^Sr by liquid scintillation counting (LSC). By using the known ^89^Sr activity and then performing spectrum deconvolution the ^90^Sr activity can be calculated. This approach is however attached with great contributions to the total combined uncertainty for high ^89^Sr/^90^Sr activity ratios, a significant increase can be observed between ratios of 10–50, which makes it unsuitable for emergency preparedness situations [17].

Previous works in this area have studied how optimizing determination of ^90^Sr via ^90^Y with regards to one sample; *n* samples (optimizing with regards to the first sample) as well as for a sequential series samples (optimizing with regards to every sample) [18–20]. In the work by Tovedal et al. [19] and the recently published first part of this work [20] ^90^Y was measured during ingrowth. Ramebäck et al. described a scenario in which ^90^Y was isolated from ^90^Sr, with regards to one sample, and measured during decay [18]. Herranz et al. published a work on optimizing the measurement time for ^89^Sr/^90^Sr determination in 2012 [21]. However, the work by Herranz presents a sub optimization seeing as it advocates awaiting full ingrowth of ^90^Y (approximately 21 days) after chemical separation.

In this work a ^90^Y measurement method is presented for a case where interference by other high energy beta radionuclides, *e.g.* ^89^Sr, other short lived strontium and yttrium radioisotopes as well as ^140^Ba/^140^La etc., complicates the Cherenkov measurement approach. The presented work gives the relationship between time allowed for ingrowth of ^90^Y, before chemical isolation of yttrium, and the allowed individual measurement time for a series of *n* samples. Important to note is that in this work the sum of all measurement times for all *n−*1 samples will also be time of decay for sample *n* (this work also takes into consideration the decay during measurement of sample *n*).

For added clarity the authors wish to define some time parameters that occur in a large extent in the paper.

- *Total analysis time* refers to the sum of the *time allowed for ingrowth* as well as the sum of all *sample measurement times*.
- *Time allowed for ingrowth* is defined as the time passed between the first separation on the resin cartridge (*i.e.* from *t* = 0 for ^90^Y) until the isolation of ^90^Y, in order to determine ^90^Y by Cherenkov counting.
- *Sample measurement time (t*_*m,n*_ *)* the time of ^90^Y measurement for any one individual sample in a sequence consisting of a total of *n* samples.
- *Time of decay (t*_*decay,n*_ *)* is the time passed between isolation of ^90^Y and the following measurement of the same radionuclide, using Cherenkov counting, for any one individual sample in a sequence consisting of a total of *n* samples.

## Theory

**Fig. 1:**
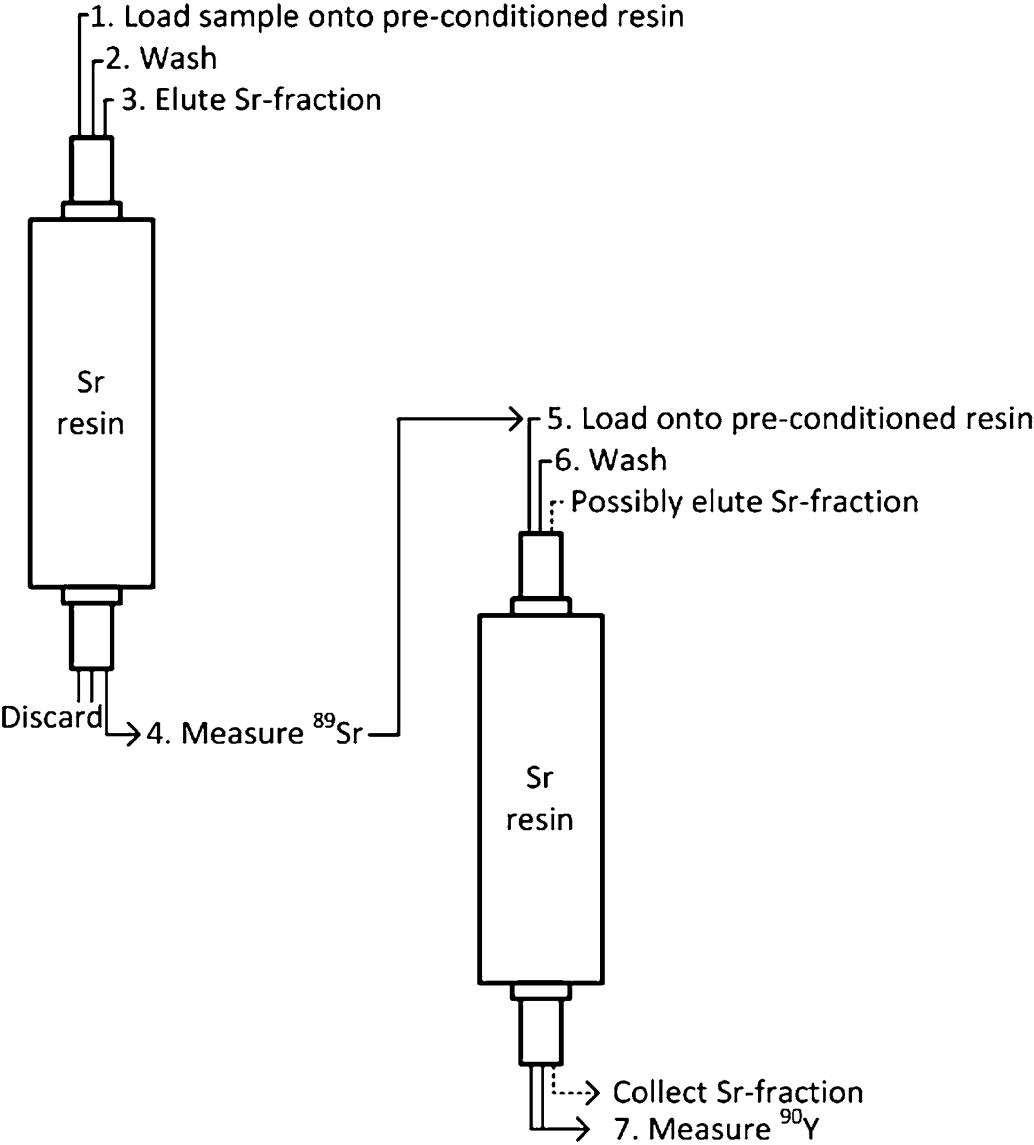
A schematic overview of the assumed chemical separation procedure for both strontium and yttrium

As in the previously published work this study was performed purely on a theoretical basis [20]. However, this work was based on the premise that both ^89^Sr and ^90^Sr are present in the sample. In order to determine ^90^Sr, by Cherenkov measurement of ^90^Y, it is necessary to chemically separate ^90^Y from ^89^Sr and ^90^Sr, this is done according to the method described in Holmgren et al. [2] as shown by Fig. 1.

### Minimal detectable activity (MDA)

As presented in the previously published work [20] the minimal detectable activity (MDA) (assumed as half the action limit for milk per sample, *i.e.* (0.5·L_A_)/V_sample_) when determining ^90^Sr, via Cherenkov measurement on ^90^Y, is expressed as;

1 $${\rm MDA}^{^{90}{\rm Sr}} = \left[ {\left( {\frac{{k^{2} + 2k\sqrt 2 \cdot \sqrt {R_{\rm b} t_{\rm m,n} } }}{{\left( {1 - e^{{ - \lambda_{\rm Y} t_{\rm m,n} }} } \right)}}} \right) \cdot \lambda_{\rm Y} } \right]\frac{{e^{{\lambda_{\rm Y} t_{\rm decay,n} }} \left( {{{\left( {e^{{\lambda_{\rm Y} t_{\rm m,n} }} - 1} \right)} \mathord{\left/ {\vphantom {{\left( {e^{{\lambda_{Y} t_{\rm m,n} }} - 1} \right)} {\lambda_{\rm Y} t_{\rm m,n} }}} \right. \kern-0pt} {\lambda_{\rm Y} t_{\rm m,n} }}} \right)}}{{\left( {\Psi \cdot U} \right)\left( {1 - e^{{ - \lambda_{\rm Y} t_{\rm ingrowth} }} } \right)}}$$

where *k* is a constant representative of a confidence interval of 95 %, *R*_b_ is the background count rate (cps), *t*_m,n_ is the measurement time (in seconds) for the sample, similarly *t*_m,n−1_ is the measurement time (in seconds) for the previous sample, *λ*_Y_ is the decay constant (s^−1^) for ^90^Y, *t*_ingrowth_ is the allowed time for ingrowth (in seconds) between step 3 and 5 in Fig. 1, *U* is the yield from the strontium separation, Ψ (cps/Bq) is the measurement efficiency of ^90^Y and *t*_decay,n_ (also in seconds) is represented by;

2 $$t_{\rm decay,n } = \left( {\mathop \sum \limits_{i}^{n} t_{\rm m, (n - 1)} } \right)$$

The results presented in this work were obtained by solving Eq. 1 with regards to *t*_m,n_ at an optimum time of ingrowth, and subsequently iterating the equation for the following samples, by means of the method presented by Dekker [22].

In this work an assumed measurement time (*t*_m_) for ^89^Sr, using Cherenkov counting, is 15 min for 1 sample (measurement efficiency of 0.37 cps/Bq). By using a modified version of Eq. 1 the MDA can be calculated according to Eq. 3;

3 $$\rm MDA_{{}}^{^{89}Sr} = {\raise0.7ex\hbox{${\left[ {\left( {\frac{{k^{2} + 2k\sqrt 2 \cdot \sqrt {R_{\rm b} t_{\rm m} } }}{{\left( {1 - e^{{ - \lambda_{\rm ^{89}{\rm Sr}} t_{\rm m} }} } \right)}}} \right) \cdot \lambda_{\rm ^{89}{\rm Sr}} } \right]}$} \!\mathord{\left/ {\vphantom {{\left[ {\left( {\frac{{k^{2} + 2k\sqrt 2 \cdot \sqrt {R_{b} t_{m} } }}{{\left( {1 - e^{{ - \lambda_{{\rm Sr}89} t_{m} }} } \right)}}} \right) \cdot \lambda_{{\rm Sr}89} } \right]} {\left( {\Psi \cdot U} \right)}}}\right.\kern-0pt} \!\lower0.7ex\hbox{${\left( {\Psi \cdot U} \right)}$}}$$

This gives a MDA of 0.11 Bq, *i.e.* a range of 0.57–57 Bq per sample, for ^89^Sr depending on the sample volume and at the in-house background count rate. This gives that the total measurement time for ^89^Sr, and a time of ^90^Y-ingrowth, of at least 2.5 h. The time needed for sample preparation as well as the separation procedure have not been taken into consideration in this work, seeing as the procedures differ greatly within the scientific community. However, for the separation method presented in Fig. 1 the time needed for separation and sample preparation can be estimated to 2.5–4 h depending on the sample volume. A rapid method for determination of strontium in milk, as described into Fig. 1, uses 5 ml of sample digested in a MARS5 microwave. Therefore this work will present most of the results for 5 mL, but it will also present some data for ranges of volumes.

### Measurement uncertainties

This work assumes that the amount of counts collected for a range of samples are the same, and therefore the uncertainty contribution should be the same as well. However, accounting for the background contribution as stated in the work by Currie and Lochamy [25, 26], for paired observations, *t*_m,n_ is equal to *t*_m,Bg_ which gives that the amount of counts in the background will increase following the additional measurement time needed to reach the set detection limit. This implies that the uncertainty will decrease slightly (according to the assumption that $unc\left( N \right) = \sqrt N$ for 1σ). Given that the uncertainty of the background measurement will be one of the primary contributors to the total combined uncertainty at MDA level activities, the total combined uncertainty is also expected to decrease somewhat with increased measurement times (all parameters can be seen in Table 1).

**Table 1:** The measurement parameters used in this work. The count rates as well as measurement efficiency are for Cherenkov counting

Parameter	Assumption	Unit
Number of samples in a series	10	Samples
Sample volume (V_sample_)	2–200	mL
^90^Sr action limit^a^	100	Bq/L
MDA per sample	0.1–10	Bq
Total chemical yield of the strontium analysis	0.5	
Measurement efficiency ^90^Y	0.65	cps/Bq
Background count-rate	0.007–0.7	cps
In-house background count rate^b^	0.0136	cps
Decay constant of ^90^Y^c^	3.006 · 10^−6^	s^−1^

^a^In milk according to WHO [23]

^b^The typical background count rate for FOI’s low background system (Wallac 1220 Quantalus, Perkin Elmer)

^c^The decay constant was calculated using the *t*_½_ (^90^Y) = 64 h [24]

## Results and discussion

**Fig. 2:**
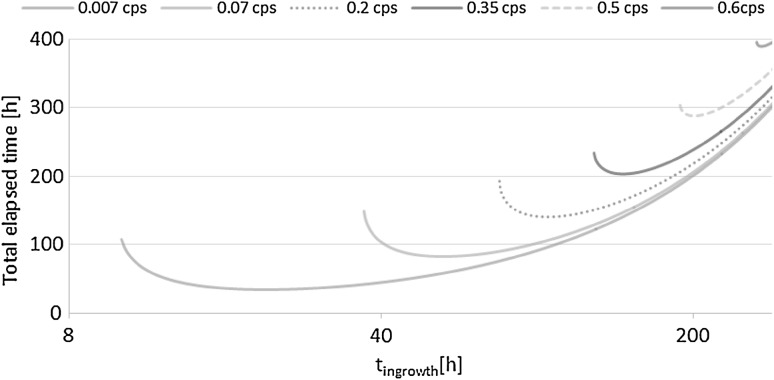
Description of how the minima for total analysis time, for ten samples in a series, changes with different background count rates. The MDA was set as constant at 0.1 Bq and *t*_ingrowth_ (steps 3–5 in Fig. 1) was varied between 2.5 h and full ingrowth of ^90^Y

Due to the separation of the mother and the daughter nuclide there will be a decreasing amount of activity within all samples (a consequence of the decay of the analyte ^90^Y). This will inevitably result in a need to extend the measurement time with each consecutive sample. To what extent the measurement time will need to be increased is dependent on the time allowed for ingrowth. An example of where to find the optimum relationship between the time allowed for ingrowth, (*t*_ingrowth_) and total analysis time can be seen in Fig. 2.

Using a low background instrument will give a less sensitive relationship between ingrowth and total analysis time that, compared to high background instruments, as illustrated in Fig. 2. For some instruments, with high backgrounds, there is no possibility to measure down to low detection limits of *e.g*. 0.1 Bq for a set amount of sample, regardless of how long the time for ingrowth is. In Table 2 it is shown that a background count rate of 0.7 cps will not yield any valid results, seeing as the last few samples in a series will always be below the MDA. If, however, the number of samples in the series is decreased from 10 to 8 it would be possible to meet the MDA criteria for all samples in the series. Table 2 also shows how the measurement time for the first and last sample in a series of ten samples differ with changing background count rates.

**Table 2:** Calculated optimized times for ingrowth and measurement, for a series of ten samples and a MDA of 0.1 Bq

Background count rate (cps)	*t*_ingrowth_ (h)	Σ(*t*_m,n_) (h)	Total analysis time (h)	Measurement time, *t*_m,n_ (h)
0.7	Full	N/A	N/A	N/A
0.35	139	65	204	3.5–13
0.07	54	28	82	2.1–3.7
0.007	22	12	34	1.1–1.3

**Fig. 3:**
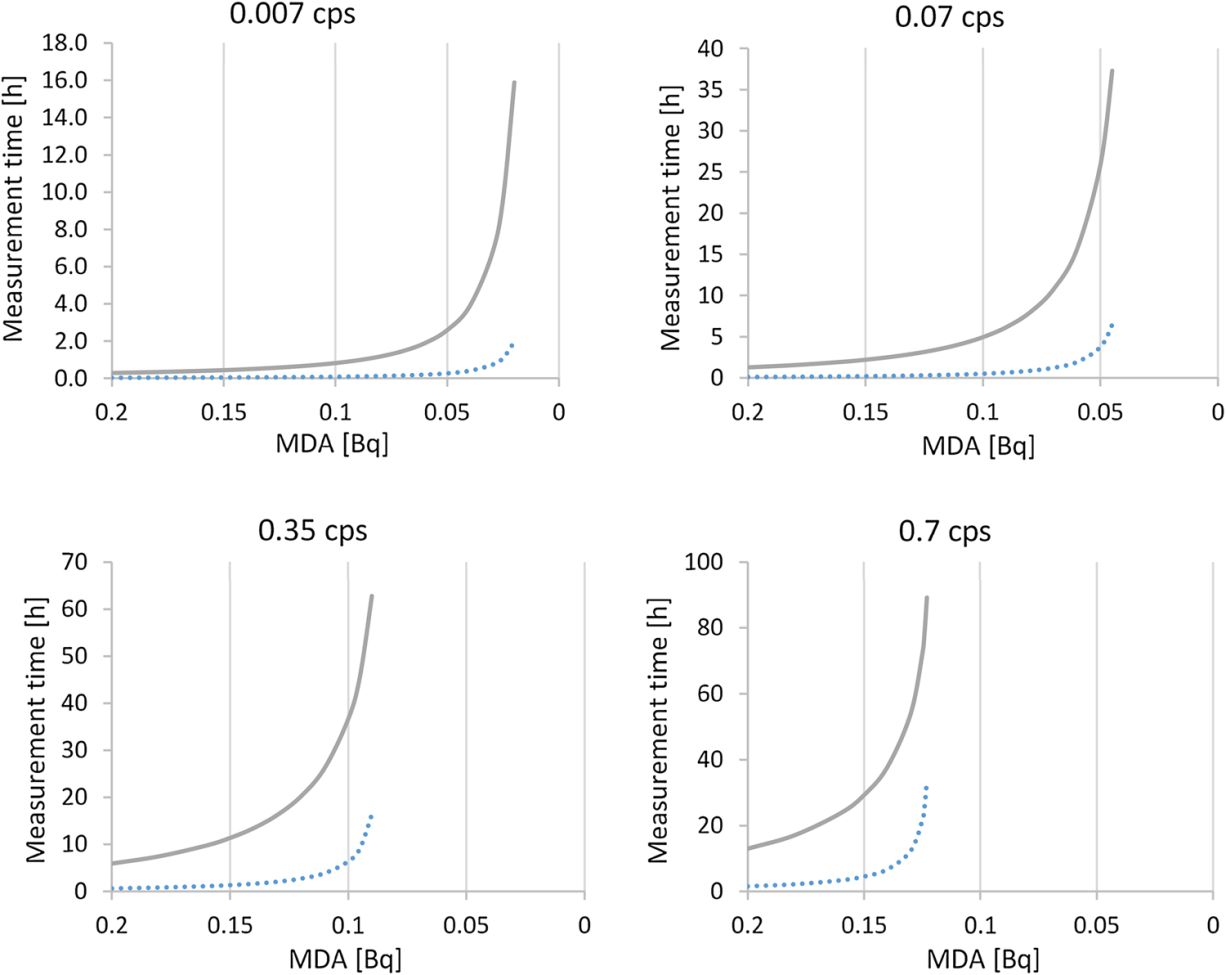
The effect on total measurement time (solid line), for a series of ten samples, and the last sample measurement (10th measurement, *dotted line*), at full ingrowth, when MDA is decreased. The background contribution for the different figures were 0.007, 0.07, 0.35 and 0.7 cps respectively

This work could also be used to identify how low it is possible to go, with regards to MDA, for a set of ten samples when ^90^Y has been allowed full ingrowth. Figure 3 shows how the total measurement time for all ten samples, as well as the individual measurement time for the 10th sample, is affected by pushing for lower MDA, with regards to different backgrounds.

Moreover, Fig. 3 also illustrates that for higher background count rates (graphs in the lower right and left hand corner) the contribution of the measurement time from the 10th sample to the total measurement time will be larger than for lower count rates. This information is helpful when choosing what volume/mass of sample to analyze (MDA is lowered with increasing volume/mass of sample), as well as what type of instrument to buy.

In order to compare the results, and also the effectiveness, of this work the calculated optimized times are put in contrast to a *standard* approach. This standard approach assumes that the individual measurement time, *t*_m,n_, is the same for each sample. And therefore the solution to Eq. 1 is solved for the 10th sample. The measurement time obtained will result in that all samples in the series will meet the MDA criterion, however, it requires longer *t*_ingrowth_.

The results presented in Table 3 show that for high MDA (5 and 10 Bq) the total analysis time is approximately the same over the given range of background count rates. But, when the MDA is low, about 1–0.1 Bq, the optimization starts to make a difference, even for lower background count rates. For a background count rate of 0.7 cps there is no measurement set up, for ten samples, which will satisfy the MDA criteria. However, using the optimized method allows up to eight samples (*t*_ingrowth_ = 283 h, *t*_m,n_ = 5 to 37 h/sample) to be measured compared to three samples (*t*_ingrowth_ = 222 h, *t*_m_ = 12 h/sample) for a standard method. An optimized measurement of eight samples might take longer than a standard measurement of three samples. But measuring six samples by the optimized method will deliver results in less than 260 h (*t*_ingrowth_ = 185 h, *t*_m,n_ = 6 to 27 h/sample), which is approximately the same time as it takes to measure three samples by a standard measurement method.

**Table 3:** The difference in measurement-, ingrowth- and total analysis time (for *n* = 10), at different MDA and backgrounds, for a standard approach and the results obtained when using the optimized approach presented in this work. All measurement times are given in hours

Method	MDA (Bq)	0.007 cps			0.07 cps			0.7 cps		
		*t*_ingrowth_	Σ(*t*_m,n_)	Total	*t*_ingrowth_	Σ(*t*_m,n_)	Total	*t*_ingrowth_	Σ(*t*_m,n_)	Total
Standard	0.1	26.1	11.4	37.5	85.6	23.0	108.6	Full	N/A	N/A
	1	4.7	2.6	7.3	10.1	4.8	14.9	24.7	10.0	34.7
	5	1.6	1.0	2.6	3.2	1.7	4.9	7.1	3.4	10.5
	10	1.0	0.7	1.7	2.0	1.1	3.1	4.3	2.1	6.5
Optimized	0.1	22.0	12.5	34.5	56.0	27.6	83.6	Full	N/A	N/A
	1	4.5	2.7	7.2	9.3	5.1	14.4	21.3	10.9	32.2
	5	1.6	1.0	2.6	3.2	1.7	4.9	6.8	3.5	10.2
	10	1.0	0.7	1.7	2.0	1.1	3.1	4.2	2.2	6.4

Furthermore, this work presents a method that allows for earlier measurement of samples compared to a standard measurement approach, *i.e.* the first results will be available for decision makers at an earlier point in time. Table 3 shows that the time saved, with regards to total analysis time, is in the range of tenths of hours for low MDA on high background instruments *e.g.* 0.1 Bq at a background of 0.07 cps.

However the greatest gain is that this method allows whole sets of samples, which will meet the MDA criteria, to be measured at higher background contributions than an un-optimized method.

## Conclusions

When performing measurements of ^90^Y with a purpose of delivering reliable ^90^Sr results above the action limit [23] it is important to consider the impact of sample volume and instrumental background in order to choose the most time efficient method. This work shows that at low background count rates in combination with a high MDA, *e.g.* when measuring large amounts of sample, the difference in total analysis time between a standard measurement method and the optimized method is negligible. Nonetheless, there is a great deal of time to save, with regards to sample throughput, for measurements at higher background count rates at low MDA, *e.g.* small amounts of samples. This implies that for small sample volumes, which generally require less time for sample preparation and strontium separation, the analysis time will be reduced significantly with this method. For a medium background count rate of 0.07 cps there is a total gain of 25 h when measuring to a MDA of 0.1 Bq. Finally, this work shows that by using an optimized measurement approach one can measure eight samples, in the same time frame as it would take to measure three by a standard measurement method, at high background count rates aiming to meet the MDA criterion of 0.1 Bq.

To conclude, the benefits of adjusting the measurement time for each individual sample in a series is most prominent when dealing with anything but low background count rates.
